# Relationship of cardiovascular disease risk and hearing loss in a clinical population

**DOI:** 10.1038/s41598-023-28599-9

**Published:** 2023-01-30

**Authors:** Rachael R. Baiduc, Joshua W. Sun, Caitlin M. Berry, Melinda Anderson, Eric A. Vance

**Affiliations:** 1 Department of Speech, Language, and Hearing Sciences, University of Colorado Boulder, 2501 Kittredge Loop Drive, 409 UCB, Boulder, CO 80309 USA; 2 Department of Applied Mathematics, University of Colorado Boulder, Boulder, CO 80309 USA; 3 Department of Otolaryngology, University of Colorado School of Medicine, Aurora, CO USA

**Keywords:** Risk factors, Epidemiology

## Abstract

Hearing loss has been associated with individual cardiovascular disease (CVD) risk factors and, to a lesser extent, CVD risk metrics. However, these relationships are understudied in clinical populations. We conducted a retrospective study of electronic health records to evaluate the relationship between hearing loss and CVD risk burden. Hearing loss was defined as puretone average (PTA_0.5,1,2,4_) > 20 dB hearing level (HL). Optimal CVD risk was defined as nondiabetic, nonsmoking, systolic blood pressure (SBP) < 120 and diastolic (D)BP < 80 mm Hg, and total cholesterol < 180 mg/dL. Major CVD risk factors were diabetes, smoking, hypertension, and total cholesterol ≥ 240 mg/dL or statin use. We identified 6332 patients (mean age = 62.96 years; 45.5% male); 64.0% had hearing loss. Sex-stratified logistic regression adjusted for age, noise exposure, hearing aid use, and body mass index examined associations between hearing loss and CVD risk. For males, diabetes, hypertension, smoking, and ≥ 2 major CVD risk factors were associated with hearing loss. For females, diabetes, smoking, and ≥ 2 major CVD risk factors were significant risk factors. Compared to those with no CVD risk factors, there is a higher likelihood of hearing loss in patients with ≥ 2 major CVD risk factors. Future research to better understand sex dependence in the hearing loss-hypertension relationship is indicated.

## Introduction

Hearing loss is estimated to affect 27.7 million adults in the USA^1^, and has been associated with depression^2^, reduced quality of life^3^, and cognitive decline^4, 5^. In older adults, hearing loss is a prevalent health condition^6^ and often presents comorbidly with other chronic conditions^7^. Once sensorineural hearing loss related to aging or systemic disease such as cardiovascular disease (CVD) begins, it cannot be reversed, making identification and treatment of modifiable risk factors critical for prevention and reduction of morbidity risk. To that end, numerous epidemiological studies have sought to identify modifiable risk factors for hearing loss with increased attention being paid to CVD risk factors (e.g. diabetes and smoking).

Various mechanisms might underlie associations between CVD risk factors and auditory dysfunction. Histological examinations have identified strial atrophy, loss of spiral ganglion neurons and outer hair cells, and basilar membrane thickening as primary pathologic findings in diabetes^8–10^. In mice, exposure to cigarette smoke leads to loss of spiral ganglion neurons, likely due to increase in cochlear oxidative stress^11^. Physiological studies in the spontaneously hypertensive rat demonstrated that the stria vascularis is the primary auditory site of lesion in hypertension^12^ and further suggest that hypertension accelerates age-related hearing loss^13^. Chinchillas fed a high-cholesterol diet have increased serum cholesterol levels, cochlear lysosomes, and accumulation of extracellular glycogen^14^. It is thought these morphological changes reflect strial and organ of Corti damage.

In human studies, reports suggest independent associations between hearing loss and tobacco smoking^15^, diabetes^16–18^, hypertension^19, 20^, and hyperlipidemia^21^. Examination of relationships with overall CVD risk have also been performed. These reports have explored associations between hearing loss and metabolic syndrome^22–26^, the American Heart Association’s Life’s Simple 7^27^, and Framingham Risk Score^28^. Results of these studies are inconclusive as some have shown that aggregate CVD risk is associated with hearing loss^22, 23, 26–28^, though others, including a longitudinal study of metabolic syndrome and incident hearing loss^24^, have not^25^. Further, most published research on this topic has utilized epidemiological datasets (e.g., National Health and Nutrition Examination Survey [NHANES]^23^, Korean NHANES^25^, Busselton Healthy Ageing Study^28^, Jackson Heart Study^27^). Published clinical reports have limited generalizability having been primarily conducted in Asian^22^ or Iranian^26^ populations. Understanding these relationships in the American clinical population is of interest because providers routinely treat patients with CVD risk factors. Using NHANES 1999–2010 data, Saydah et al.^29^ found that ~ 70% of U.S. adults ≥ 18 years have at least one CVD risk factor with multiple comorbidities being common.

Sex differences factor into both hearing and cardiovascular health. For example, evidence suggests that women with certain CVD risk factors (e.g., diabetes) are at higher risk of CVD events (e.g. stroke) than men^30^. Male sex has been associated with hearing loss in humans^31^ and reduced cochlear integrity in rhesus monkeys^32^. There is a higher risk of incident hearing loss among men, even for low-risk men (e.g. thin, educated, non-smoker)^33^. Some studies relating hearing loss to CVD risk factors suggest sex-specific associations. A significant relationship between self-reported myocardial infarction and cochlear impairment was observed in the Epidemiology of Hearing Loss Study, but only in women^34^. A study of Medicare beneficiaries in the Health, Aging, and Body Composition study found that high blood pressure (BP) was associated with hearing loss, but only in white men^35^. An investigation of persons > 80 years old found the association between CVD risk factors and hearing loss to be stronger in men, a finding partly explained by cardioprotective effects of estrogen^36^. Together this evidence suggests that there may be sex-based differences in hearing status related to CVD-risk factors although significant gaps in our understanding remain.

The aim of the present retrospective chart review was to investigate the relationship between hearing loss and CVD risk factor burden in a clinical sample. Our assessment of CVD risk was based on risk factors commonly managed in the primary care setting; namely, hypertension, hyperlipidemia, tobacco smoking, and diabetes. Adjusting for potential confounders, we compared hearing in patients with major CVD risk factors to hearing in patients with optimal CVD risk status (i.e. no major risk factors). A secondary aim was to evaluate sex differences in these relationships. We hypothesized that increasing CVD risk load would be associated with greater odds of hearing loss. In doing so, we sought to advance understanding of the relationship between CVD risk and hearing loss, which may illuminate pathways for improved prevention and intervention.

## Materials and methods

### Study design and setting

We conducted a retrospective review of clinical data from patients seen at UCHealth, a large Colorado academic medical center, between 1-Jan 2011 and 31-Jul 2019. Patients aged ≥ 18 years who underwent audiological and general health evaluations (including BP measurement and glucose and lipid panels) were identified via query of Health Data Compass Data Warehouse electronic health records (EHRs).

### Chart review

We manually extracted audiological data from the records of 7069 patients, which were entered into a deidentified database. We directly exported all other data including patient demographics and CVD risk factor status from EHRs. Identifiable personal health information was not included in the database. Ethical approval and informed consent statements were exempted (#19-1400) by the Colorado Multiple Institutional Review Board. All methods were carried out in accordance with relevant guidelines and regulations.

**Figure 1:**
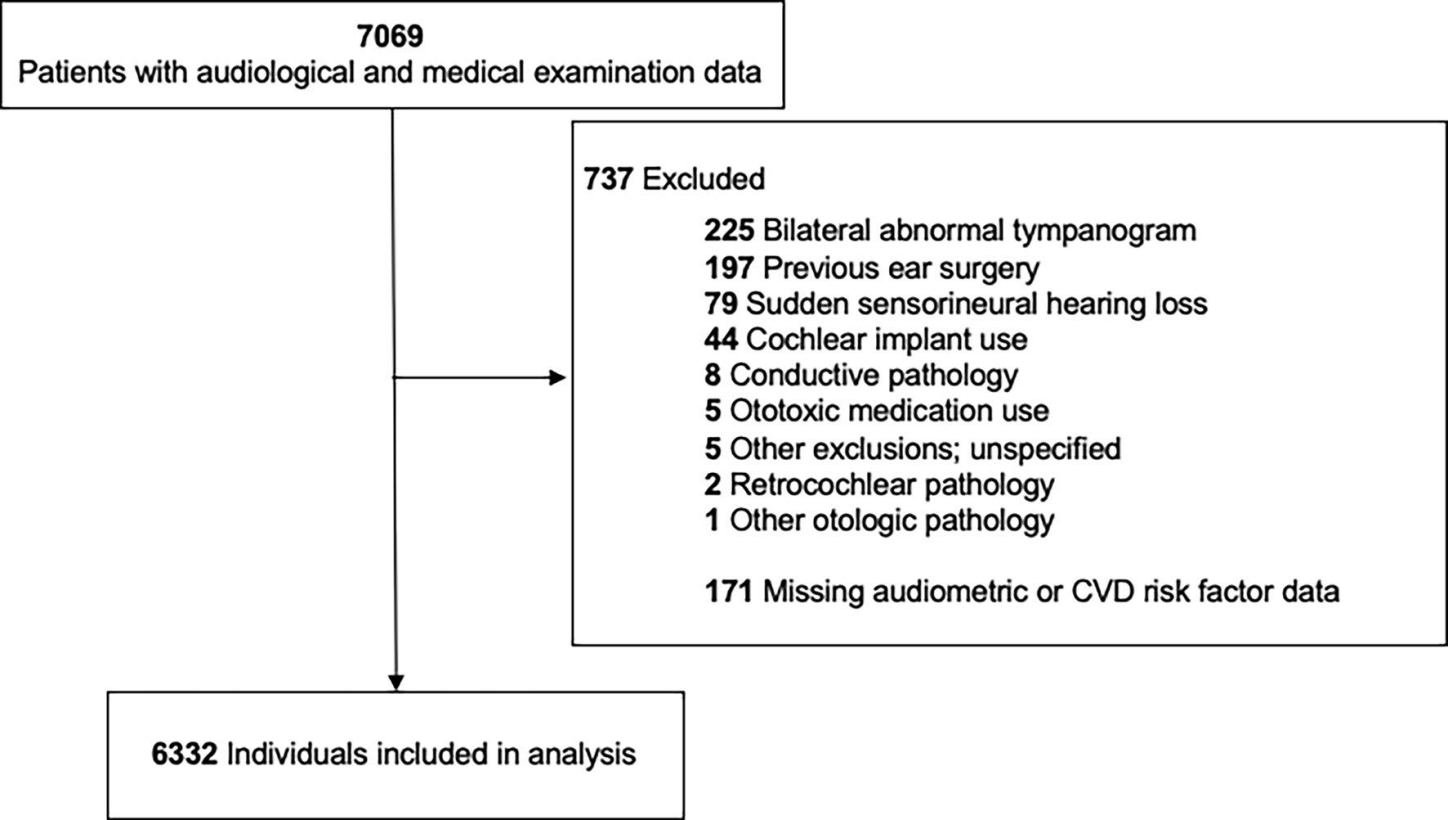
Flowchart of patient exclusions.

From the core group of patients, we excluded individuals with bilateral abnormal tympanograms (i.e. static acoustic admittance < 0.3 or > 1.8 mmohs or peak pressure < − 150 daPa), suspected or diagnosed conductive pathology (primarily identified by measurement of an air–bone gap > 10 dB at one or more frequencies between 250 and 4000 Hz), sudden sensorineural hearing loss, history of otologic surgery, acoustic neuroma, cochlear implants, ototoxic medication use, and missing CVD risk factor or audiometric data resulting in a final sample of 6332 patients (Fig. 1).

### Determination of cardiovascular disease risk status

Diabetes, tobacco smoking, hyperlipemia, hypertension served as independent variables. We classified tobacco smoking status (Never/Current/Former) based on self-report. We defined diabetes as use of antihyperglycemics, fasting plasma glucose ≥ 126 mg/dL, random serum/plasma glucose ≥ 200 mg/dL [or 11.1 mmol/L]), and/or physician diagnosis indicated by International Classification of Disease codes (versions 9 and 10). We categorized BP into four strata based on systolic (S) and diastolic (D) BP levels: [a] SBP ≥ 160 mmHg or DBP ≥ 100 mmHg, or use of antihypertensives, [b] SBP 140–159 or DBP 90–99 mmHg (elevated), [c] SBP 120–139 or DBP 80–89 mmHg (not optimal), and [d] SBP < 120 and DBP < 80 mmHg (optimal; reference group)^37^. We defined four strata for total cholesterol: [a] ≥ 240 mg/dL or statin use, [b] 200–239 mg/dL (elevated), [c] 180–199 mg/dL (not optimal), and [d] < 180 mg/dL (optimal; reference group). Diabetes, current smoking, cholesterol group (a), and BP group (a) were classified as major risk factors.

Based on the four CVD risk factors, we defined CVD risk burden categorically and continuously. First, per Bogle et al.^37^ we created five mutually exclusive risk categories: [a] optimal levels of all four CVD risk factors, [b] ≥ 1 CVD risk factors non-optimal, [c] ≥ 1 CVD risk factors elevated, [d] exactly 1 major CVD risk factor, and [e] ≥ 2 major CVD risk factors. Second, we computed Framingham Risk Scores per D’Agostino et al.’s algorithm^38^. This score represents 10-year absolute CVD risk and risk of individual events (e.g. heart failure, peripheral arterial disease, etc.).

### Audiological assessment and outcome variables

Audiological data consisted of information obtained during audiological examination (i.e. otoscopy, tympanometry, and air and bone conduction audiometry). Audiologic evaluations were completed by a licensed audiologist based on our institution’s standard of care. We captured portions of the assessment, including case history, and air- and bone-conduction puretone audiometry (Air: 0.5, 1, 2, 3, 4, 6, 8 kHz; Bone: 0.5, 1, 2, 4 kHz). If necessary, masking was performed, and masked thresholds were used in place of unmasked thresholds in statistical models. For all frequencies, in the case of nonresponse, thresholds were defined as 130 dB HL. Finally, noise exposure history (recreational, non-military occupational, and military) was determined via case history. For statistical analysis, noise history was collapsed into one binary variable (Yes/No).

Our primary outcome variable was hearing loss, which we defined as average threshold at 0.5, 1, 2, and 4 kHz (hereafter, puretone average [PTA_0.5,1,2,4_]) > 20 dB HL in the poorer ear. We also evaluated hearing thresholds continuously at discrete audiometric frequencies.

### Statistical analysis

We report continuous measures as mean (SEM) and categorical measures as number (percent). We compared categorical variables using the Chi-square test and continuous variables with two-tailed *t*-tests. We used logistic regression to determine associations between hearing loss and CVD risk (four individual risk factors and overall risk). We used linear regression to compare thresholds at individual audiometric frequencies between the five CVD risk strata using the optimal group as reference. We also used linear regression to examine the relationship between PTA_0.5,1,2,4_ and Framingham Risk Score. Fully adjusted models included age, sex (unless stratified), noise exposure, hearing aid use (yes/no), and body mass index (BMI) as covariates. Models for individual risk factors (e.g. diabetes) were adjusted for the other three CVD risk factors. We fit models separately for men and women. Additional models were constructed for prediabetes and former tobacco smoking although these factors were not considered for aggregate CVD risk classification. P-values < 0.05 were considered statistically significant. Analysis was done using the glm function in R (R Core Team (2019) v. 3.6.1).

### Conference presentation

Portions of this work were presented at the Association of Diabetes Care and Education Specialists conference in August of 2020 and at the American Academy of Audiology conference in April of 2021.

## Results

We present patient characteristics in Table 1. We identified 4052 patients with hearing loss. Individuals with hearing loss were older than those without hearing loss (68.75 [SEM = 0.19] vs. 52.67 [SEM = 0.28] years; *p* < 0.001). The hearing loss group contained more males than the normal hearing group (49.85% vs. 37.68%, respectively; *p* < 0.001). For both groups, most individuals were non-Hispanic white. High cholesterol, hypertension, and diabetes were more common in patients with hearing loss; the prevalence of current smoking was similar between groups.

**Table 1:** Patient demographic and cardiovascular characteristics by hearing status.

	Hearing loss (N = 4052)*		Normal hearing (N = 2280)		*P* value
	Mean or %	SEM or *N*	Mean or %	SEM or *N*	
Demographic variables					
Sex, male	49.85%	2020	37.68%	859	< 0.001
Age, years	68.75	0.19	52.67	0.28	< 0.001
Race					
American Indian and Alaska Native	0.32%	13	0.18%	4	< 0.001
Asian	3.58%	145	2.72%	62	
Black or African American	6.24%	253	7.24%	165	
Multiple race	0.81%	33	1.14%	26	
Native Hawaiian and other Pacific Islander	0.10%	4	0.00%	0	
Other	6.71%	272	6.40%	146	
Unknown	0.74%	30	2.28%	52	
White or Caucasian	81.49%	3302	80.04%	1825	
Ethnicity					
Hispanic	6.42%	260	7.06%	161	0.016
Non-Hispanic	92.30%	3740	90.70%	2068	
Patient refused	0.59%	24	0.83%	19	
Unknown	0.69%	28	1.40%	32	
Cardiovascular variables					
BMI (kg/m^2^)	27.86	0.09	27.36	0.13	0.001
Total cholesterol (mg/dL)	177.31	0.66	185.33	0.82	< 0.001
Use of cholesterol lowering medication	43.02%	1743	18.29%	417	< 0.001
Cholesterol strata (mg/dL)					
< 180	22.88%	927	35.09%	800	< 0.001
180–199	11.77%	477	17.24%	393	
200–239	17.47%	708	22.11%	504	
≥ 240 or statin use	47.88%	1940	25.57%	583	
Systolic blood pressure (mm Hg)	130.90	0.30	124.98	0.37	< 0.001
Diastolic blood pressure (mm Hg)	74.20	0.16	75.15	0.22	< 0.001
Use of antihypertensives	29.15%	1181	13.25%	302	< 0.001
Blood pressure strata (mm Hg)^†^					
SBP < 120 and DBP < 80	19.97%	809	34.43%	785	< 0.001
SBP 120–139 or DBP 80–89	31.24%	1266	35.70%	814	
SBP 140–159 or DBP 90–99	15.28%	619	13.42%	306	
BP ≥ 160 or DBP ≥ 100, or use of antihypertensives	33.51%	1358	16.40%	374	
Estimated average glucose (mg/dL)^‡^	128.53	0.60	117.91	0.60	< 0.001
Glucose tolerance fasting (mg/dL)^‡^	102.43	6.19	108.80	3.32	0.387
Glucose serum/plasma (mg/dL)^‡^	109.72	0.68	98.90	0.65	< 0.001
Glucose random serum/plasma (mg/dL)^‡^	106.08	0.67	97.44	0.84	< 0.001
Use of antihyperglycemics	15.70%	636	6.23%	142	< 0.001
Diabetes					
No	20.06%	813	31.45%	717	< 0.001
Yes	36.85%	1493	19.52%	445	
Prediabetes	43.09%	1746	49.04%	1118	
Smoking					
Never	53.33%	2161	68.29%	1557	< 0.001
Current	5.70%	231	4.78%	109	
Former	40.97%	1660	26.93%	614	
CVD risk burden					
All risk factors optimal^§^	4.15%	168	12.72%	290	< 0.001
≥ 1 risk factors not optimal^‖^	10.61%	430	20.26%	462	
≥ 1 risk factors elevated^¶^	14.19%	575	21.84%	498	
1 major risk factor^#^	32.97%	1336	28.46%	649	
≥ 2 major risk factors**	38.08%	1543	16.71%	381	
Framingham risk score^‡,††^	0.21	0.00	0.09	0.00	< 0.001

P-values from Chi-square tests (categorical variables) or *t* tests (continuous variables).

*BMI* body mass index, *DBP* diastolic blood pressure, *PTA* pure-tone average *SBP* systolic blood pressure, *SEM* standard error of the mean.

*Defined as PTA_0.5,1,2,4_ > 20 dB HL in the worse ear.

^†^One patient was excluded from logistic regression models due to missing BP group status.

^‡^N for hearing loss and normal hearing groups, respectively. Estimated average glucose: N = 2944, 1465; Glucose tolerance fasting: N = 7, 54; Glucose serum/plasma: N = 3824, 2113; Glucose random serum/plasma: N = 3251, 1576; Framingham Risk Score: N = 5452 overall.

^§^All CVD RFs optimal: BP < 120 and < 80 mmHg, total cholesterol < 180 mg/dL, not currently smoking, and no diabetes.

^‖^ ≥ 1 CVD RF not optimal: BP 120–139 or 80–89 mmHg or total cholesterol 180–199 mg/dL, not currently smoking, and no diabetes.

^¶^ ≥ 1 CVD RFs elevated: 140–159 or 90–99 mmHg, or total cholesterol 200–239 mg/dL, not currently smoking, and no diabetes.

^#^Exactly 1 major CVD RF: SBP ≥ 160 or DBP ≥ 100 mmHg or on treatment, or cholesterol ≥ 240 mg/dL or on treatment, or diabetes, or current tobacco smoking.

** ≥ 2 major CVD RFs: having at least 2 RFs from footnote #.

^††^Defined per D’Agostino et al. (2008).

Table 2 shows noise exposure history and average hearing sensitivity (PTA_0.5,1,2,4_) by hearing loss status. Noise exposure, particularly military-related, was more common in persons with hearing loss than those without. For those with hearing loss, most (84.35%) had bilateral loss and on average, hearing loss was mild (worse ear PTA_0.5,1,2,4_ of 39.83 dB HL [SEM = 0.26]). Supplementary Table S1 reports the distribution of hearing loss by CVD risk status and sex. For both sexes, the prevalence of hearing loss generally increased as CVD risk load increased. For each risk strata, there were more males with hearing loss than females. This difference was greatest in the lowest risk stratum wherein 54.86% (95% CI 47.18–62.32) of males, but only 25.44% (95% CI 20.56–31.01) of females, had hearing loss.

**Table 2:** Audiological characteristics by hearing status.

Characteristic	Hearing loss (N = 4052)*		No hearing loss (N = 2280)	
	Mean or %	SEM or *N*	Mean or %	SEM or *N*
Audiological data				
Noise exposure, yes	39.17%	1587	28.64%	653
Recreational	9.97%	404	11.32%	258
Non-military occupational	12.22%	495	8.95%	204
Military	10.98%	445	4.43%	101
Unknown/type not reported	10.37%	420	6.40%	146
Puretone average,^†^ dB HL				
Better ear	33.83	0.22	11.18	0.09
Worse ear	39.83	0.26	13.50	0.09
Hearing loss better ear, yes	84.35%	3418	0.00%	0.00
Hearing loss poorer ear, yes	100.00%	4116	0.00%	0.00

*dB HL* decibels hearing level, *SEM* standard error of the mean.

*Defined as PTA_0.5,1,2,4_ > 20 dB HL in the worse ear.

^†^Defined as average threshold at 0.5, 1, 2, 4 kHz.

**Figure 2:**
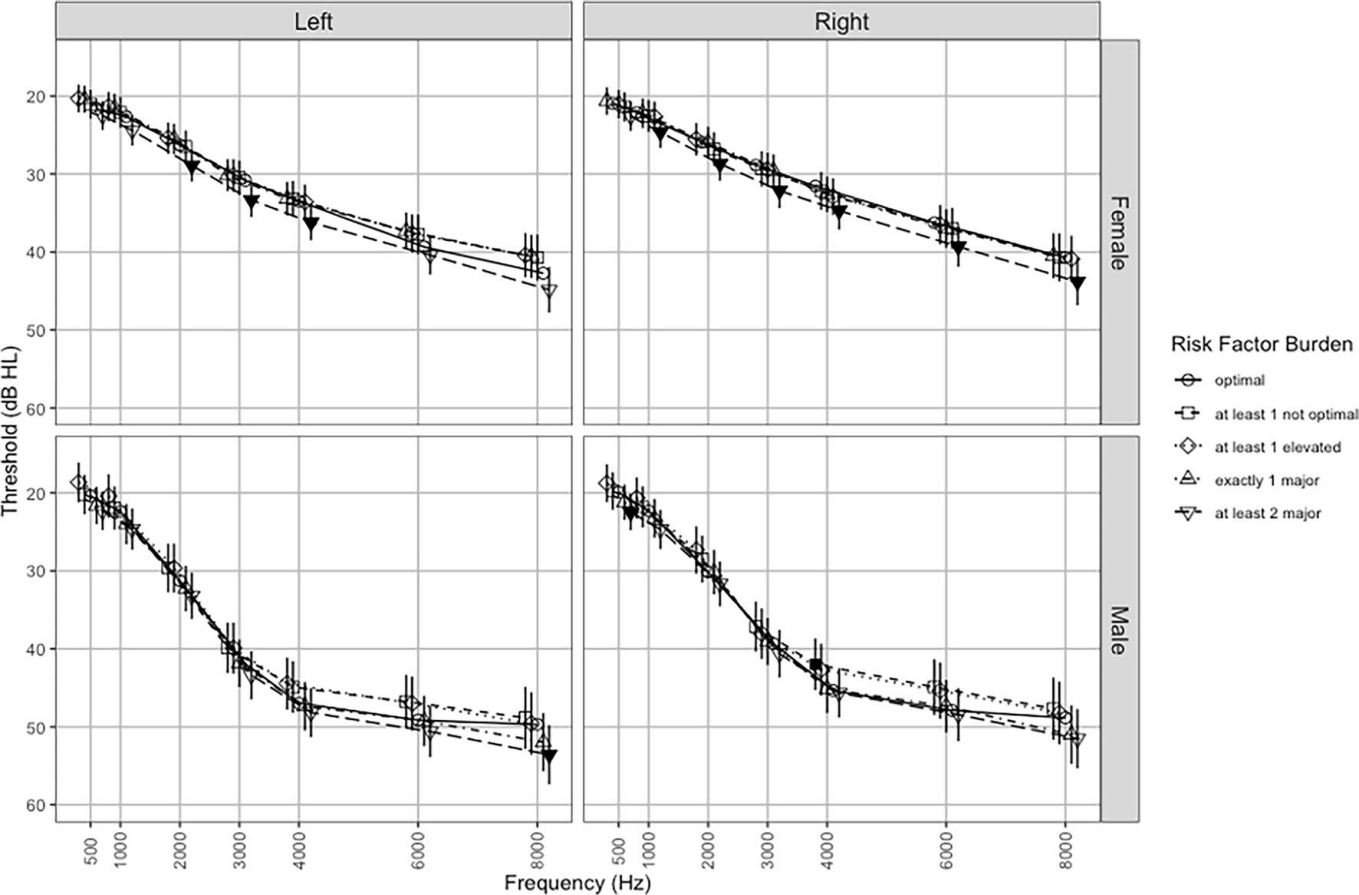
Sex-stratified audiograms^*^ (mean [95% CI] by ear and CVD risk group. Filled symbols indicate *P* < 0.05 in comparison to optimal CVD (reference group). Data points jittered on x-axis to provide greater visual clarity of error bars. *adjusted for age, hearing aid use, BMI, noise exposure.

Supplementary Table S2 reports unadjusted hearing thresholds which tended to increase (worsen) with increasing CVD risk load. On average, hearing sensitivity was 7.27 dB worse in men than women. Sex-stratified ear-specific audiograms adjusted for age, BMI, hearing aid use, and noise exposure are shown in Fig. 2. Males had more severe and more steeply sloping hearing loss than females. In males, those with ≥ 1 non-optimal CVD risk factor had significantly better hearing at 4.0 kHz (right ear) and those with ≥ 2 major CVD risk factors had poorer hearing at 0.5 kHz (right ear) and 8.0 kHz (left ear) vs. the optimal risk group. In females, compared to the optimal risk group, those with ≥ 2 major CVD risk factors had significantly poorer hearing at all frequencies between 1.0 and 8.0 kHz (right ear) and at 2.0, 3.0 and 4.0 kHz (left ear).

Associations between hearing loss and CVD risk factors are shown in Table 3, which includes age-sex and multivariable-adjusted models. The age-sex adjusted model shows that diabetes, hypertension (specifically, BP group [d]), current and former smoking, and having 1 or ≥ 2 major CVD risk factors were associated with hearing loss. Except former tobacco smoking and hypertension, the significance of these associations was retained in the fully adjusted model. The strongest relationships were those with current smoking (OR = 2.02 [95% CI 1.52–2.71]) and ≥ 2 major CVD risk factors (OR = 2.23 [95% CI 1.67–3.00]). The odds of hearing loss increased with increasing BP and total cholesterol levels, and with increasing CVD risk.

**Table 3:** Age-sex^*^ and multivariable adjusted† odds ratios (95% CI) for hearing loss^‡^.

	Age-sex* adjusted		Multivariable^†^ adjusted	
	OR (95% CI)	*P* value	MVOR (95% CI)	*P* value
Diabetes				
No diabetes	*1.0 [Ref]*		*1.0 [Ref]*	
Diabetes	1.55 (1.29, 1.86)	< 0.001	1.58 (1.30, 1.93)	< 0.001
Prediabetes	0.95 (0.81, 1.11)	0.518	0.96 (0.81, 1.13)	0.624
Blood pressure (mm Hg)				
Optimal, SBP/DBP, < 120/ < 80	*1.0 [Ref]*		*1.0 [Ref]*	
SBP, 120–139 or DBP 80–89	1.05 (0.90, 1.23)	0.525	1.01 (0.85, 1.21)	0.881
SBP 140–159 or DBP 90–99	1.13 (0.92, 1.38)	0.236	1.11 (0.89, 1.38)	0.365
SBP/DBP, ≥ 160/ ≥ 100 or medication use	1.24 (1.03, 1.5)	0.020	1.20 (0.98, 1.47)	0.078
Total cholesterol (mg/dL)				
Optimal, < 180	*1.0 [Ref]*		*1.0 [Ref]*	
180–199	1.00 (0.82, 1.23)	0.962	0.98 (0.79, 1.21)	0.829
200–239	1.11 (0.93, 1.33)	0.258	1.11 (0.91, 1.34)	0.305
≥ 240 mg/dL (or statin use)	1.14 (0.96, 1.34)	0.128	1.13 (0.95, 1.35)	0.175
Tobacco smoking				
Never	*1.0 [Ref]*		*1.0 [Ref]*	
Current	1.93 (1.47, 2.56)	< 0.001	2.02 (1.52, 2.71)	< 0.001
Former	1.18 (1.03, 1.35)	0.016	1.11 (0.96, 1.28)	0.165
CVD risk factor burden^§^				
All RF optimal	*1.0 [Ref]*		*1.0 [Ref]*	
≥ 1 risk factor not optimal	1.18 (0.89, 1.56)	0.250	1.20 (0.89, 1.63)	0.241
≥ 1 risk factor elevated	1.26 (0.96, 1.65)	0.097	1.31 (0.98, 1.76)	0.069
1 major risk factor	1.48 (1.14, 1.91)	0.003	1.44 (1.09, 1.91)	0.011
≥ 2 major RF	2.19 (1.68, 2.86)	< 0.001	2.23 (1.67, 3.00)	< 0.001

Significant values are in italics.

*DBP* diastolic blood pressure, *MVOR* multivariable adjusted odds ratio, *SBP* systolic blood pressure.

*All CVD risk factors are covariates in individual risk factor models.

^†^Adjusted for age, sex, noise exposure, hearing aid use, and BMI. Individual risk factors are covariates in individual risk factor models.

^‡^Defined as PTA_0.5,1,2,4_ > 20 dB HL in the worse ear.

^§^Defined as per Table 1.

**Figure 3:**
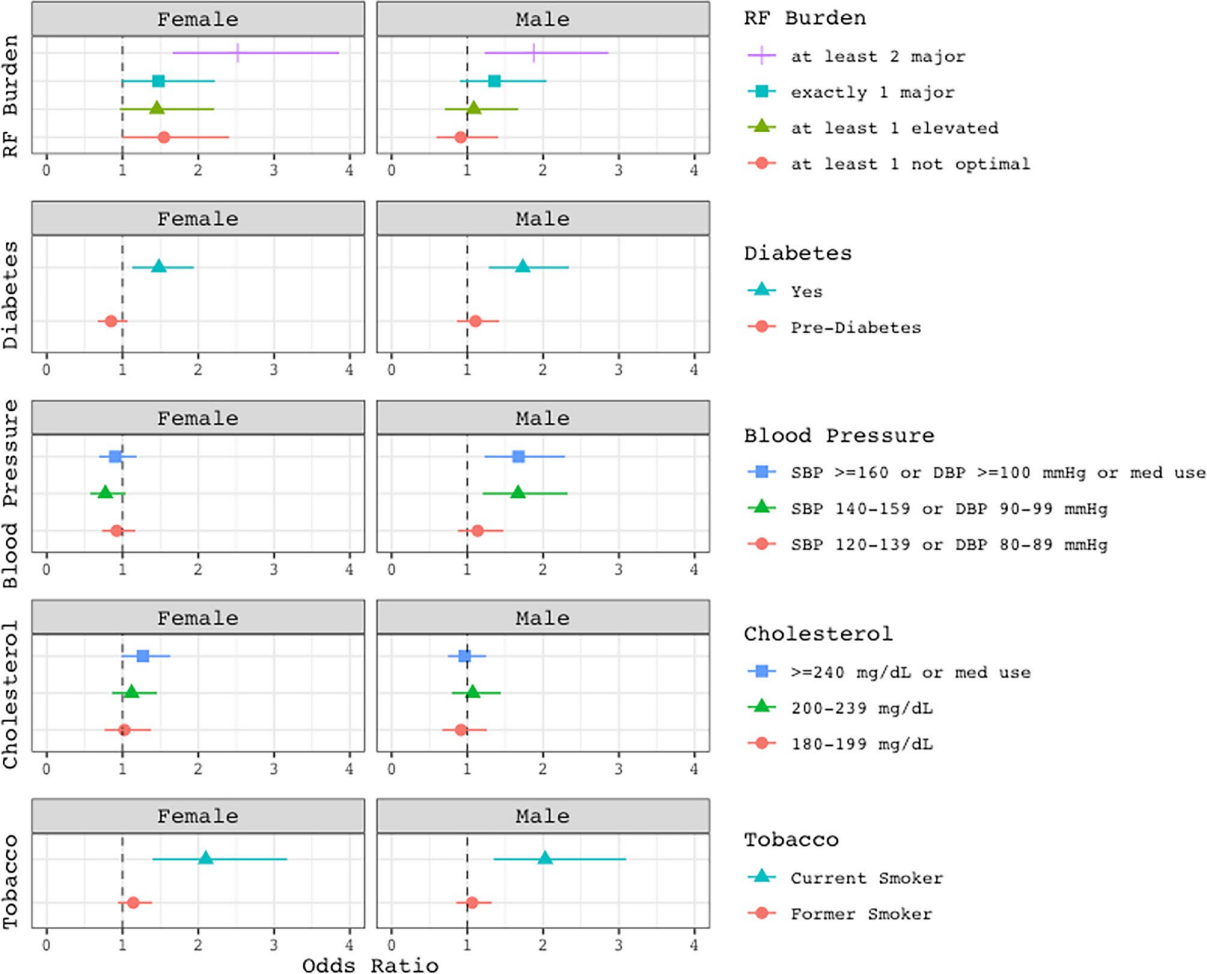
Sex-stratified forest plots of multivariable adjusted odds ratios^*^ (95% CI) for hearing loss^†^. *DBP* diastolic blood pressure, *RF* risk factor, *SBP* systolic blood pressure. * Adjusted for age, noise exposure, hearing aid use, and BMI. Individual risk factors are covariates in individual risk factor models. † Defined as PTA_0.5,1,2,4_ > 20 dB HL in the worse ear.

Next, because sex-specific relationships were identified upon audiometric analysis (Fig. 2), we report sex-stratified multivariable adjusted ORs (MVOR) for hearing loss in Fig. 3. Amongst females, diabetes, current smoking, and having ≥ 2 major CVD risk factors were significantly associated with increased odds of hearing loss (MVOR = 1.48; [95% CI 1.13–1.94]; MVOR = 2.10 [95% CI 1.40–3.17]; MVOR = 2.52 [95% CI 1.66–3.86], respectively). Similarly, in males, estimated associations of hearing loss with diabetes, smoking, and ≥ 2 major CVD risk factors were as follows: MVOR = 1.73 (95% CI 1.28–2.34), MVOR = 2.03 (95% CI 1.35–3.10), and MVOR = 1.88 (95% CI 1.23–2.86), respectively. High BP was also associated with hearing loss with MVOR = 1.67 (95% CI 1.20–2.32) and MVOR = 1.68 (95% CI 1.23–2.29) for BP groups (b) and (a), respectively.

Finally, the strength of the relationship between Framingham Risk Score and PTA_0.5,1,2,4_ is reflected by the* R* value of 0.419 shown in Supplementary Fig. S1, suggesting that as risk increased, hearing sensitivity worsened.

## Discussion

This study evaluated the relationship between CVD risk factors and hearing loss in 6332 patients. We used CVD risk metrics commonly employed in primary care and hypothesized that there would be a positive relationship between increasing CVD risk factor load and likelihood of hearing loss. First, our results demonstrated that diabetics and current smokers had increased odds of hearing loss. Having ≥ 2 major CVD risk factors was associated with 92% increased odds of hearing loss compared to the optimal CVD risk stratum. Second, hypertension was associated with hearing loss, but only in males. Other relationships were similar between the sexes.

Of the CVD risk factors, the strongest relationship we observed was that with current smoking, which was associated with hearing loss in both sexes (MVOR = 1.85 [95% CI: 1.40–2.46]). Our findings are consistent with cross-sectional reports from the Epidemiology of Hearing Loss Study^15^, Korean NHANES^39^, and the UK Biobank Resource^40^, which utilized a speech-in-noise test. Prospective research has also linked smoking to hearing loss^41^. In our study, former smoking was not associated with hearing loss, which has been shown previously^40^. Former smokers may adopt other positive lifestyle changes (e.g. improved diet, physical activity, BP control), which could be protective for auditory health. Smoking cessation may mitigate the excess risk of hearing loss associated with smoking, although further research is needed to corroborate this possibility.

Diabetics were at 43% higher odds of hearing loss than non-diabetics. The strength of this association was similar between the sexes. The link we observed with diabetes is consistent with past reports^8, 16, 17, 42^. In a meta-analysis, Akinpelu et al.^16^ reported a slightly more robust association with diabetes than we found. Based on six studies, they calculated an average OR for hearing loss of 1.91 (95% CI 1.47–2.49). The studies they evaluated defined hearing loss using a cut point of 25 dB HL whereas we used 20 dB HL. Other factors such as diabetic control, disease duration, and participant demographics could also account for variable strength of the reported associations.

Previous studies on the relationship between hearing loss and hyperlipemia are inconclusive and report contradictory results^21, 25, 43–45^. We did not find evidence to support an association between high cholesterol and hearing loss. There was widespread use of statins in our sample. In patients with hearing loss, 43% were on statins. Moreover, 18% of individuals with normal hearing were on treatment. A cross-sectional analysis of Blue Mountain Hearing Study participants aged ≥ 50 years found cholesterol-lowering medication to be negatively associated with hearing loss^21^. One contested possibility is that treatment positively influences auditory status, which may obscure any negative influence of hyperlipidemia itself. However, a randomized trial of the effect of atorvastatin on hearing loss progression did not find evidence to support this hypothesis^46^.

The one sex-specific relationship we observed was that between hearing loss and hypertension, which was limited to males. The prevalence of hypertension was 42% overall (45% in males and 39% in females; *data not shown*). Cross-sectional reports including those from the NHANES^31^, Busselton Healthy Ageing Study^28^, and Hispanic Community Health Study/Study of Latinos^47^ have identified significant relationships between hypertension and hearing loss. Sex differences have also been reported. For example, a study of 3315 participants (aged 52–99 years) from the Rotterdam Study reported a significant relationship between low-frequency hearing loss and systolic BP in men, but not women^48^. An association between hypertension and high-frequency hearing loss was reported in men, but not women, from the Framingham cohort^49^. Wattamwar et al.^36^ observed a stronger relationship between CVD risk factors and hearing loss in elderly males than females. The underpinning(s) of these sex differences is unknown but may relate to the otoprotective effects of estrogen, which has been proposed for age-related and noise-induced hearing loss^50^.

Results of this study extend the current literature on CVD comorbidities and hearing loss. Our multivariate model showed that having ≥ 1 major CVD risk factors significantly increases odds of hearing loss. This is important because most individuals had ≥ 1 major CVD risk factors, with hyperlipidemia being the most common. Of the patients with hearing loss, the majority (71%) had ≥ 1 major risk factors while only 45% of individuals with normal hearing had ≥ 1 major risk factors (Table 1). In the overall sample, we observed a graded association between CVD risk and likelihood of hearing loss with MVORs for exactly 1 and ≥ 2 major risk factors being 1.44 (95% CI 1.09–1.91) and 2.23 (95% CI 1.67–3.00), respectively. We also observed a correlation between PTA and Framingham Risk Score (Supplementary Fig. S1).

We observed dose-dependent relationships between odds of hearing loss and BP and total cholesterol levels, but these associations were not significant in the overall sample. Our findings indicate that increasing risk factor load is associated with greater likelihood of hearing loss. However, this seems to be the case only for major CVD risk factors as non-optimal risk factor status (e.g. prediabetes) was not significantly related to hearing loss. Our approach to defining CVD risk differs somewhat from prior reports. Nonetheless, comparisons to past research can be made. With an average participant age similar to ours, Tan et al.^28^ identified a graded association between Framingham Risk Score and hearing loss in the Busselton Healthy Ageing Study. Sun et al.^23^ found a higher number of metabolic syndrome components to be related to hearing loss in the NHANES and a separate NHANES study determined diabetes and smoking to be a particularly harmful combination^51^.

The clinical implications of this work are relevant to medical practitioners across specialties. Early identification of auditory dysfunction in persons with modifiable risk factors such as smoking is critical to reducing the disease burden of hearing loss. Knowledge that certain risk factors and high-risk factor load are associated with hearing loss may guide recommendations for hearing evaluations and intervention for at-risk patients. With increased attention to personalized medicine and patient-centered care, these findings support holistic medical care. In the future, clinical implications will be better understood once prospective studies can address how baseline CVD risk status influences long term auditory function.

This retrospective study has important limitations. The cross-sectional design hampers determinations regarding causality and progression of hearing loss. In some instances, we were unable to capture CVD comorbidity status on the day of audiological evaluation as visits for hearing and other health concerns did not always coincide. We used CVD risk factor and BMI data from the date of the closest clinical encounter and cannot account for potential day-to-day variability in these measures. We did not adjust for medication use as the definition of CVD risk stratification included treatment. Our audiometric analysis did not adjust for multiple hypothesis tests. Data from primarily white cohorts were used to develop the Framingham Risk Score algorithm. It may not provide precise CVD risk estimates for all racial/ethnic groups. Data used to develop the original algorithm were from subjects aged 30–74 years whereas patients in our dataset ranged from 18–97 years. Last, we were only able to calculate risk scores for 5452 patients as calculation requires information that was not available for all individuals (namely, high-density lipoprotein levels).

In conclusion, this study provides data on hearing status as it relates to adverse cardiovascular health. Diabetes, smoking, and the presence of ≥ 2 major CVD risk factors significantly increased the odds of hearing loss. In males, hypertension was also a significant predictor. Improved glucose control, smoking cessation, and early BP management (for males) may promote healthy hearing although such strategies would need to be borne out by longitudinal studies.

## Supplementary Information

Supplementary Figure S1.

Supplementary Tables.

## Data Availability

All relevant data are within the paper and tables/figures. Raw data that support the findings of this study are available from the corresponding author upon reasonable request.
