# Supplementary figures and images for: Relationship of cardiovascular disease risk and hearing loss in a clinical population
Source: Sci Rep. 2023 Jan 30;13:1642. doi: 10.1038/s41598-023-28599-9 (PMC9886989; doi:10.1038/s41598-023-28599-9)

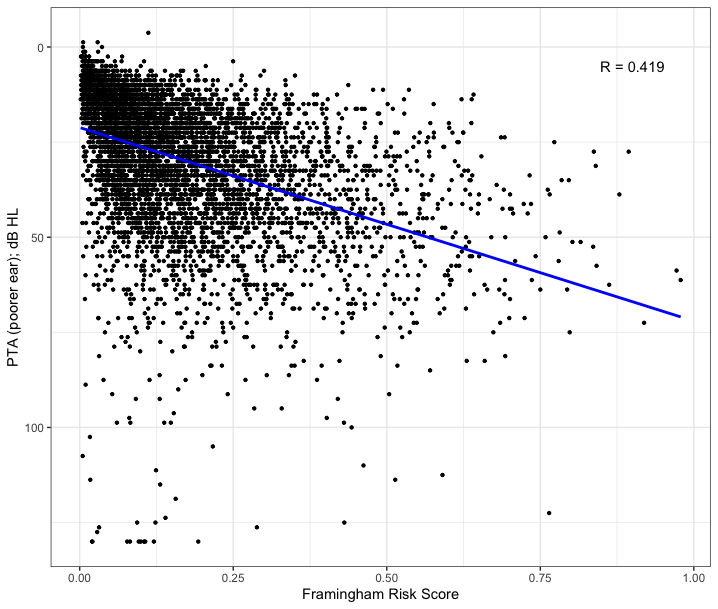

Supplement: Supplementary file 1 — Supplementary Figure S1. [file 41598_2023_28599_MOESM1_ESM.png]
