# Supplementary material for: Relationship of cardiovascular disease risk and hearing loss in a clinical population
Source: Sci Rep. 2023 Jan 30;13:1642. doi: 10.1038/s41598-023-28599-9 (PMC9886989; doi:10.1038/s41598-023-28599-9)
Supplement: Supplementary file 2 — Supplementary Tables. [file 41598_2023_28599_MOESM2_ESM.docx]

**SUPPLEMENTAL TABLE AND FIGURE LEGENDS**

**Supplementary Table S1. Distribution of hearing loss^*^ (poorer ear) by CVD risk factor burden.**

^*^ Defined as PTA_0.5,1,2,4_ >20 dB HL in the worse ear

† Defined as per Table 1

**Supplementary Table S2. Sex-stratified unadjusted mean (SEM) thresholds (dB HL) by frequency (Hz) for each CVD risk strata**

Abbreviations: CVD, cardiovascular disease; SEM, standard error of the mean

**Supplementary Figure S1. Scatter plot of PTA (dB HL) vs. CVD risk score^*^**

Abbreviations: dB HL, decibels hearing level; PTA, puretone average

^*^ risk score computed as per D’Agostino et al. (2008)

**Supplemental Table S1**

| **Hearing Loss by CVD Risk Burden Strata^†^** | | | | | |
| --- | --- | --- | --- | --- | --- |
|  | **All Risk Factors Optimal** | **≥ 1 Risk Factors Not Optimal** | **≥ 1 Risk Factors Elevated** | **1 Major Risk Factor** | **≥ 2 Major Risk Factors** |
| Overall, N | 168 | 430 | 575 | 1336 | 1543 |
| % (95% CI) | 36.68 (32.29, 41.30) | 48.21 (44.89, 51.54) | 53.59 (50.55, 56.60) | 67.30 (65.18, 69.36) | 80.20 (78.33, 81.94) |
| Female, N | 72 | 195 | 340 | 689 | 736 |
| % (95% CI) | 25.44 (20.56, 31.01) | 43.24 (38.63, 47.96) | 52.07 (48.61, 55.95) | 62.24 (59.30, 65.09) | 76.75 (73.92, 79.36) |
| Male, N | 96 | 235 | 235 | 647 | 807 |
| % (95% CI) | 54.86 (47.18, 62.32) | 53.29 (48.51, 58.01) | 55.95 (51.05, 60.74) | 73.69 (70.62, 76.55) | 83.63 (81.11, 85.88) |

**Supplemental Table S2**

| **CVD Risk Factor Burden Strata** | **Frequency, Hz** | **Mean (SEM)** | ***N*** | **Mean (SEM)** | ***N*** | **Mean (SEM)** | ***N*** |
| --- | --- | --- | --- | --- | --- | --- | --- |
|  |  | **MEN** | | **WOMEN** | | **OVERALL** | |
| All Risk Factors Optimal | 500 | 20.26 (1.37) | 175 | 15.51 (0.84) | 283 | 17.33 (0.74) | 458 |
| ≥ 1 Risk Factor Not Optimal | 500 | 19.34 (0.79) | 441 | 19.14 (0.65) | 451 | 19.24 (0.51) | 892 |
| ≥ 1 Risk Factor Elevated | 500 | 17.69 (0.57) | 420 | 21.10 (0.67) | 653 | 19.77 (0.47) | 1073 |
| Exactly 1 Major Risk Factor | 500 | 24.17 (0.60) | 878 | 23.53 (0.46) | 1107 | 23.81 (0.37) | 1985 |
| ≥ 2 Major Risk Factors | 500 | 26.39 (0.56) | 965 | 27.72 (0.54) | 959 | 27.05 (0.39) | 1924 |
| All Risk Factors Optimal | 1000 | 21.97 (1.52) | 175 | 15.85 (0.89) | 283 | 18.19 (0.81) | 458 |
| ≥ 1 Risk Factor Not Optimal | 1000 | 20.74 (0.88) | 441 | 19.78 (0.73) | 451 | 20.25 (0.57) | 892 |
| ≥ 1 Risk Factor Elevated | 1000 | 19.18 (0.68) | 420 | 22.42 (0.71) | 653 | 21.15 (0.51) | 1073 |
| Exactly 1 Major Risk Factor | 1000 | 26.98 (0.67) | 878 | 25.32 (0.51) | 1107 | 26.05 (0.41) | 1985 |
| ≥ 2 Major Risk Factors | 1000 | 29.25 (0.63) | 965 | 30.48 (0.60) | 959 | 29.86 (0.43) | 1924 |
| All Risk Factors Optimal | 2000 | 29.71 (1.85) | 175 | 18.34 (0.96) | 283 | 22.69 (0.96) | 458 |
| ≥ 1 Risk Factor Not Optimal | 2000 | 27.04 (1.05) | 441 | 23.92 (0.84) | 451 | 25.47 (0.67) | 892 |
| ≥ 1 Risk Factor Elevated | 2000 | 26.58 (0.95) | 420 | 25.91 (0.76) | 653 | 26.17 (0.60) | 1073 |
| Exactly 1 Major Risk Factor | 2000 | 35.97 (0.80) | 878 | 30.15 (0.58) | 1107 | 32.72 (0.48) | 1985 |
| ≥ 2 Major Risk Factors | 2000 | 39.14 (0.69) | 965 | 35.45 (0.63) | 959 | 37.30 (0.47) | 1924 |
| All Risk Factors Optimal | 3000 | 37.79 (2.08) | 170 | 20.07 (1.05) | 277 | 26.81 (1.10) | 447 |
| ≥ 1 Risk Factor Not Optimal | 3000 | 35.25 (1.17) | 434 | 26.07 (0.94) | 440 | 30.63 (0.76) | 874 |
| ≥ 1 Risk Factor Elevated | 3000 | 35.80 (1.16) | 412 | 29.63 (0.80) | 645 | 32.03 (0.67) | 1057 |
| Exactly 1 Major Risk Factor | 3000 | 45.61 (0.84) | 869 | 33.93 (0.62) | 1091 | 39.11 (0.52) | 1960 |
| ≥ 2 Major Risk Factors | 3000 | 49.74 (0.70) | 959 | 39.70 (0.67) | 946 | 44.75 (0.50) | 1905 |
| All Risk Factors Optimal | 4000 | 43.51 (2.11) | 175 | 21.91 (1.12) | 283 | 30.16 (1.17) | 458 |
| ≥ 1 Risk Factor Not Optimal | 4000 | 40.38 (1.23) | 441 | 28.47 (1.01) | 451 | 34.36 (0.82) | 892 |
| ≥ 1 Risk Factor Elevated | 4000 | 40.18 (1.19) | 420 | 34.03 (0.88) | 653 | 36.43 (0.71) | 1073 |
| Exactly 1 Major Risk Factor | 4000 | 51.90 (0.85) | 878 | 38.07 (0.66) | 1107 | 44.18 (0.55) | 1985 |
| ≥ 2 Major Risk Factors | 4000 | 55.59 (0.71) | 965 | 43.65 (0.69) | 959 | 49.64 (0.51) | 1924 |
| All Risk Factors Optimal | 6000 | 44.99 (2.29) | 171 | 24.75 (1.28) | 279 | 32.44 (1.27) | 450 |
| ≥ 1 Risk Factor Not Optimal | 6000 | 41.13 (1.31) | 437 | 31.51 (1.13) | 445 | 36.28 (0.88) | 882 |
| ≥ 1 Risk Factor Elevated | 6000 | 41.94 (1.25) | 417 | 36.65 (0.95) | 649 | 38.72 (0.76) | 1066 |
| Exactly 1 Major Risk Factor | 6000 | 53.06 (0.93) | 872 | 41.89 (0.73) | 1103 | 46.82 (0.59) | 1975 |
| ≥ 2 Major Risk Factors | 6000 | 57.73 (0.78) | 962 | 48.04 (0.77) | 953 | 52.91 (0.56) | 1915 |
| All Risk Factors Optimal | 8000 | 43.36 (2.60) | 174 | 24.89 (1.46) | 283 | 31.93 (1.40) | 457 |
| ≥ 1 Risk Factor Not Optimal | 8000 | 41.80 (1.53) | 436 | 32.60 (1.32) | 448 | 37.14 (1.02) | 884 |
| ≥ 1 Risk Factor Elevated | 8000 | 42.67 (1.42) | 419 | 39.78 (1.14) | 650 | 40.91 (0.89) | 1069 |
| Exactly 1 Major Risk Factor | 8000 | 55.78 (1.06) | 876 | 45.56 (0.84) | 1104 | 50.08 (0.67) | 1980 |
| ≥ 2 Major Risk Factors | 8000 | 61.77 (0.89) | 957 | 53.99 (0.88) | 956 | 57.88 (0.63) | 1913 |
